# Informed acceptance and perceptions of the 2025 ILAE seizure classification following structured teaching

**DOI:** 10.1002/epd2.70278

**Published:** 2026-05-11

**Authors:** Sándor Beniczky, Maša Malenica, Nicola Lawrence, Victoria San Antonio‐Arce, Jessica Norvell, J. Helen Cross, Eugen Trinka

**Affiliations:** ^1^ Department of Clinical Neurophysiology Danish Epilepsy Centre Filadelfia Dianalund Denmark; ^2^ Copenhagen University Hospital, Rigshospitalet, Member of the European Reference Network EpiCARE Copenhagen Denmark; ^3^ Department of Clinical Medicine University of Copenhagen Copenhagen Denmark; ^4^ Department of Child Neurology, Sestre Milosrdnice UHC, Member of the European Reference Network EpiCARE University of Zagreb Zagreb Croatia; ^5^ Great Ormond Street Hospital for Children NHS Foundation Trust London UK; ^6^ Hôpital Sant Joan de Déu, Member of European Reference Network on Rare and Complex Epilepsies EpiCARE Barcelona Spain; ^7^ Freiburg Epilepsy Center, Medical Center – University of Freiburg, Faculty of Medicine University of Freiburg Freiburg im Breisgau Germany; ^8^ International League Against Epilepsy Washington DC USA; ^9^ BRC NIHR Great Ormond Street Institute of Child Health University College London London UK; ^10^ Department of Neurology, Neurocritical Care and Neurorehabilitation, Christian Doppler University Hospital, Centre of Cognitive Neuroscience Paracelsus Medical University Salzburg Austria; ^11^ Member of EpiCARE, AustriaNeuroscience Institute, Christian Doppler University Hospital, Centre of Cognitive Neuroscience Paracelsus Medical University Salzburg Austria; ^12^ Karl Landsteiner Institute for Clinical Neuroscience Salzburg Austria

**Keywords:** consciousness, education, EpiCARE, epilepsy, ILAE, seizure classification, survey, webinar

## Abstract

**Objective:**

To assess informed acceptance and perceptions of the 2025 update of the International League Against Epilepsy (ILAE) seizure classification—after participants had received a focused educational introduction to the updated classification.

**Methods:**

We analyzed anonymized live poll responses from two educational webinars dedicated to the updated seizure classification: an EpiCARE webinar held on March 26, 2026, and an ILAE e‐Forum held on April 15, 2026. At the start of the webinars, participants reported their professional background and prior familiarity with the updated classification. Each webinar then included a 20‐min teaching session, after which participants answered a poll question on their opinion of the updated classification. In the EpiCARE webinar, participants additionally classified 16 representative video‐EEG cases using the updated classification. For the case exercise, we summarized question‐level accuracy, the most frequent incorrect answer, the micro‐average accuracy (total number of correct responses across all submitted answers divided by the total number of submitted answers), and the macro‐average accuracy (mean of the 16 individual case accuracies).

**Results:**

A total of 323 participants actively engaged in the two webinars (185 in the EpiCARE webinar and 138 in the ILAE e‐Forum). Most respondents were specialist neurologists or pediatric neurologists/pediatricians. Before the teaching session, most respondents had heard about the updated classification but had not read the full position paper and practical guide. The main post‐teaching poll was completed by 295 participants (91.3%). The vast majority of the respondents (242; 82%) rated the updated classification as feasible and useful, 17 (5.8%) as feasible but not useful, and 2 (0.7%) as not feasible, and 34 (11.5%) respondents stated they were unsure / had no opinion. In the case‐based exercise on applying the updated classification, the most frequently selected answer was correct in all 16 cases. The micro‐average accuracy was 74.3%, the macro‐average accuracy 74.5%, and the median question‐level accuracy 81.5% (range 30.4%–94.0%).

**Significance:**

When neurologists and other professionals were surveyed after structured teaching, acceptance of the 2025 ILAE seizure classification was strongly favorable. These findings suggest that education is likely to influence how the update is perceived and may help explain part of the skepticism reported in earlier surveys conducted without a standardized teaching intervention.

**Key points**

- Acceptance of the 2025 ILAE seizure classification was assessed after a focused online teaching session.
- 323 participants from 87 countries, mainly neurologists and pediatricians, actively engaged in the webinars.
- After teaching, 82% rated the updated classification as feasible and useful.
- Most participants correctly classified seizures in 16 representative video‐EEG cases.
- Structured education improves acceptance and practical use of the updated ILAE seizure classification.

## 1 INTRODUCTION

In 2025, the ILAE published an updated classification of epileptic seizures, building on the operational framework introduced in 2017.^1^, ^2^, ^3^ The update preserved the four main seizure classes—focal, generalized, unknown whether focal or generalized, and unclassified—while introducing refinements intended to improve clarity, translatability, and real‐world usability across diverse clinical settings.^1^, ^2^, ^3^ Among the most discussed changes were the replacement of awareness with consciousness as a classifier for focal seizures and seizures of unknown origin, the operational definition of consciousness through both recall and responsiveness, the introduction of a basic versus expanded version of seizure descriptors, consisting of observable versus non‐observable manifestations in the basic description, and the chronological sequence of seizure semiology in the expanded version.^1^, ^2^, ^3^

The updated classification emerged in response to implementation experience with the 2017 version. The ILAE working group cited difficulties with translation of awareness into several languages, concerns about how well the 2017 terminology mapped onto ordinary neurological practice, and the need for a classification that could function both in resource‐limited settings and in specialized epilepsy centers.^1^, ^2^, ^3^ A subsequent prospective multicenter study from secondary referral centers reported that all seizures in 458 consecutive patients could be classified with the basic version of the updated classification, and that the information needed to assess consciousness and observable manifestations could be obtained in nearly all patients.^4^ Another study found that persons with epilepsy and caregivers were generally able to understand and apply a brief patient‐friendly explanation of ictal impaired consciousness.^5^ Together, these reports support the practical intent of the updated version.

At the same time, the acceptance of the update among healthcare professionals has been the subject of debate. A survey of Spanish‐speaking neurologists and neurology residents reported mixed views of the updated classification presented to them in English language: although many respondents agreed that the 2017 version should be updated, many also felt that the 2025 revision came too soon, and the 2017 version remained the preferred framework overall.^6^ In a subsequent letter, methodological concerns were raised regarding the sampling strategy, the language of the survey materials, and, most importantly, the possibility that some respondents had limited exposure to the updated classification when expressing their opinion.^7^ This critique is important because acceptance of a new classification may depend not only on its content but also on whether clinicians have been given sufficient information, context, and examples to understand how the system is meant to be used.

Education was explicitly anticipated as part of the implementation strategy of the 2025 update.^2^, ^3^ Yet, to our knowledge, there are limited data on how clinicians and other professionals perceive the updated seizure classification, when given sufficient information, after a structured educational introduction. We therefore aimed to assess acceptance and perceptions of the 2025 ILAE seizure classification following focused teaching delivered during two international webinars. In addition, in one of the webinars, we evaluated how accurately participants applied the updated classification to representative video‐EEG cases.

## 2 METHODS

We conducted an observational study of anonymized webinar poll responses obtained during two educational webinars devoted to the updated ILAE seizure classification. Both webinars were delivered in English. The first webinar was organized under the auspices of the European Reference Network for Epilepsy (EpiCARE) on March 26, 2026. The second was organized by the ILAE as an e‐Forum on April 15, 2026, entitled “Definitions – how do they work in epilepsy?”.^8^ Both webinars were advertised through organizational newsletters and social media channels.

At the beginning of each webinar, participants were asked about their professional background and their prior familiarity with the updated seizure classification. Each webinar then included a 20‐min teaching session on the 2025 update. Following the teaching session, participants were asked the main opinion question: “What is your opinion of the updated seizure classification?” Responses were collected anonymously through the webinar polling platform.

The EpiCARE webinar also included an interactive “Classify this!” segment comprising 16 representative video‐EEG cases. Participants viewed seizure recordings and classified each case according to the updated seizure classification. For each case, we recorded the proportion of correct responses and the most frequently selected incorrect response. We summarized overall performance using two complementary measures. The micro‐average accuracy was calculated as the total number of correct responses across all submitted answers for all 16 cases divided by the total number of submitted answers. The macro‐average accuracy was calculated as the arithmetic mean of the 16 question‐level accuracies, thus weighting each case equally regardless of the number of respondents to that question.

The present report is descriptive. Categorical variables are summarized as counts and percentages. Continuous summaries for the case exercise are reported as the mean, median, minimum, maximum, and interquartile range of the question‐level accuracies. Because the objective was to evaluate the informed perception and acceptance, rather than the effect of the educational intervention per se, we did not poll the participants before the session due to the risk that emotionally motivated responses before the educational session may be carried forward to the post‐education responses.

## 3 RESULTS

A total of 323 persons, from 87 countries (Figure S1), actively participated in the two webinars: 185 in the EpiCARE webinar and 138 in the ILAE e‐Forum. The professional background of the participants is shown in Table 1. The largest groups were specialist neurologists and specialist pediatric neurologists/pediatricians (28.5% and 23.2% respectively), although the webinars also included residents, other specialists, and participants categorized as “other.”

**TABLE 1:** Professional background of the participants.

Professional background	Number of respondents (%)
Specialist—Neurologist	92 (28.5%)
Specialist—Pediatric neurologist/Pediatrician	75 (23.2%)
Specialist—Other	25 (7.7%)
Resident—Neurologist	29 (9.0%)
Resident—Pediatric neurologist/Pediatrician	14 (4.3%)
Resident—Other	4 (1.2%)
Other	84 (26.0%)

Of the 323 participants, 295 (91.3%) responded to the polls concerning the updated seizure classification. Participants were asked how familiar they were with the updated seizure classification before the teaching session (Table 2). Among the 295 respondents to this question, most had heard about the update but had not read the full position paper and practical guide (56.3%), whereas 38.3% had read both papers. Only 5.4% reported that they had not heard of the update before the lecture.

**TABLE 2:** Level of familiarity with the updated seizure classification before the webinar.

Response	Number of respondents (%)
I have heard of it, but have not read the full papers	166 (56.3%)
I have read both the position paper and the practical guide	113 (38.3%)
I have not heard of it before this lecture	16 (5.4%)

Of the 295 respondents who rated the updated seizure classification after the educational session, 242 (82.0%) considered the classification feasible and useful, 17 (5.7%) considered it feasible but not useful, 2 (0.8%) considered it not feasible, and 34 (11.5%) stated that they were unsure or had no opinion (Table 3). Thus, the overwhelming majority of respondents viewed the update favorably after the teaching session.

**TABLE 3:** Perceptions and acceptance of the updated ILAE seizure classification (2025).

Response	Number of respondents (%)
Feasible And useful	242 (82.0%)
Unsure/No opinion	34 (11.5%)
Feasible, but not useful	17 (5.8%)
Not feasible	2 (0.7%)

The case‐based classification exercise on applying the updated seizure classification from the EpiCARE webinar is summarized in Table 4. For all 16 cases, the most frequently selected answer was correct. The overall correct response rate (micro‐average) was 74.3%, and the average correct response rate (macro‐average) was 74.5%. The median question‐level accuracy was 81.5%, with a minimum of 30.4%, a maximum of 94.0%, and an interquartile range of 64%–87%.

**TABLE 4:** Performance in the EpiCARE “Classify this!” exercise (16 representative cases). Accuracy was evaluated at the level of the expanded classification because participants were shown video‐EEG recordings.

Case	Seizure type (descriptor)	Correct answers (%)	Most frequent incorrect answer—%
1	Myoclonic seizure	45.5%	Epileptic spasm—25.7%
2	Focal Seizure (Focal myoclonic jerk)	58.2%	Myoclonic seizure—20.6%
3	Epileptic spasm	86.0%	Generalized tonic–clonic seizure—2.5%
4	Focal Seizure (Focal epileptic spasm)	65.2%	Focal preserved consciousness seizure—16.8%
5	Tonic seizure	71.0%	Generalized seizure (not further specified)—16.8%^a^
6	Atonic seizure	86.9%	Negative myoclonic seizure—4.6%
7	Negative myoclonic seizure	77.1%	Myoclonic‐atonic seizure—5.6%
8	Typical absence seizure	88.3%	Atypical absence seizure—3.9%
9	Atypical absence seizure	93.3%	Generalized seizure (not further specified)—2%^a^
10	Myoclonic absence seizure	60.4%	Clonic seizure—10.7%
11	Eyelid myoclonia with/without absence	94.0%	Generalized seizure (not further specified)—2%^a^
12	Generalized tonic–clonic seizure	75.3%	Generalized seizure (not further specified)—9.3%^a^
13	Focal Impaired Consciousness Seizure (Epigastric aura → oro‐alimentary automatism → partially responsive → unresponsive + gestural automatism (R) + head‐deviation (R))	86.1%	Unknown whether focal or generalized, impaired consciousness seizure—4.6%
14	Focal Preserved Consciousness Seizure (Integrated hypermotor automatism → gyratory to the right)	30.4%	Unknown whether focal or generalized, preserved consciousness seizure—22.3%
15	Focal Preserved Consciousness Seizure (Somatosensory aura‐R foot → Jacksonian propagation)	86.3%	Unknown whether focal or generalized, preserved consciousness seizure—6.2%
16	Focal‐to‐Bilateral Tonic–Clonic Seizure (Vocalization → Ictal cry → Focal tonic—R face →Figure‐of‐four—extension: R‐elbow → bilateral tonic→bilateral clonic)	88.7%	Focal impaired consciousness seizure—4.7%

^a^ In these cases, the most frequent incorrect answer would have been acceptable at the level of the basic classification. However, scoring was performed at the level of the expanded classification because participants were shown video‐EEG recordings.

## 4 DISCUSSION

This study was designed to evaluate the informed acceptance and perceptions of the 2025 ILAE seizure classification after participants had received a focused educational introduction to the update. The principal finding was that post‐teaching acceptance was strongly favorable: more than four‐fifths of respondents judged the updated classification feasible and useful. In addition, the interactive case‐based exercise on the application of the updated classification showed that webinar participants were able to apply the updated framework with reasonably high overall accuracy across a diverse set of seizure types and semiological patterns.

These findings should be interpreted against the backdrop of the recent debate on the updated classification. In the earlier survey of neurologists and residents, opinions were mixed and the 2017 version remained the preferred classification overall.^6^ The subsequent commentary highlighted a key concern: opinions about a newly published classification may be difficult to interpret if respondents have had limited exposure to the underlying rationale, terminology, and practical examples.^7^ Our findings address that concern directly. By assessing perceptions only after a structured teaching session, this study suggests that at least part of the resistance to the 2025 update may reflect incomplete familiarity rather than a settled rejection of the classification itself.

The favorable post‐teaching response is also consistent with the implementation logic of the 2025 update. The revision was intentionally framed as an operational refinement rather than a wholesale replacement of the previous classification and the 2017 framework.^1^, ^2^, ^3^ The ILAE working group emphasized greater translatability, alignment with established neurological language, and usability across different resource settings.^1^, ^2^, ^3^ The strong positive response in our webinars suggests that these aims become more visible once the changes are explained explicitly. Education may be especially important for aspects that have generated debate, including the reintroduction of consciousness as a classifier, the combined use of responsiveness and recall to operationally define consciousness, and the distinction between observable and non‐observable manifestations.^1^, ^2^, ^3^, ^4^, ^5^

The case‐based component provides an additional perspective. The most frequently selected answer was correct in all 16 cases, and the overall accuracy was in the mid‐70% range despite the brevity of the teaching session and the evaluation at the granularity of the expanded version. This supports the practical learnability of the updated seizure classification and suggests that clinicians can apply it with good accuracy after concise instruction. The lower accuracy for selected focal cases, especially those requiring a more nuanced interpretation within the expanded classification, is also informative. It indicates where future teaching materials may be most valuable: not in the basic structure of the classification, but in subtler distinctions relevant to semiology, level of description, and the interface between basic and expanded classification.

The findings of our study also support the view that updates in seizure terminology should not rely on publication alone, but should be accompanied by active and structured dissemination, as emphasized in a recent commentary paper: “the updated seizure classification should be paired with a robust dissemination strategy to ensure that these updates reach their target audience and help improve the treatment of seizures.”.^9^ Our results are consistent with this perspective. Before the teaching sessions, most participants had heard of the updated classification but had not read the full background papers, whereas after a brief 20‐min educational intervention the large majority considered the updated classification feasible and useful. This suggests that acceptance of the 2025 ILAE seizure classification is influenced not only by the content of the classification itself, but also by whether clinicians are given clear, practical, and accessible opportunities to learn how to apply it. In this regard, webinars, case‐based exercises, concise summaries, and other point‐of‐care educational tools may have a key role in accelerating informed uptake of the updated terminology across diverse clinical settings.

Our study has limitations. First, the sample was self‐selected and consisted of webinar attendees; therefore, the findings may not be generalizable to all neurologists or to all professionals involved in epilepsy care. Individuals who attend epilepsy education webinars may already be more motivated, more engaged with classification issues, or more open to innovation than the broader target population. Second, this was an immediate post‐teaching assessment, so we cannot determine whether favorable perceptions would persist over time or translate into durable changes in daily clinical documentation. Third, the main perception outcome was based on a single opinion item, which captures an overall practical judgment but does not separate conceptual agreement, usability, and personal preference. Finally, due to the anonymous polling, we did not have individual‐level linked data allowing subgroup analyses by profession, prior familiarity, or webinar type.

Despite these limitations, the study has notable strengths. It addresses a concrete gap identified in the early debate on the 2025 update, namely the need to assess perceptions after respondents have been informed about the new framework. It also combines attitudinal polling with a practical classification exercise, thereby linking perceived usefulness with real‐time application. In this respect, the study complements the growing implementation literature showing that the updated classification is feasible in secondary referral settings^4^ and understandable to patients and caregivers when properly explained.^5^

In conclusion, structured teaching was associated with strong acceptance of the 2025 ILAE seizure classification in two international webinars, and participants were able to apply the framework with high accuracy in representative cases. These findings support the view that education is not peripheral to implementation of the updated classification; it is likely one of its key determinants. Future studies should examine retention of these attitudes over time and determine which educational strategies most effectively support consistent adoption in routine clinical practice.

## AUTHOR CONTRIBUTIONS

Sándor Beniczky: conceptualization, design, data collection, data analysis and interpretation, drafting the manuscript. Maša Malenica: data collection, data analysis and interpretation, editing the manuscript. Nicola Lawrence, Victoria San Antonio Arce, Jessica Norvell, J. Helen Cross: data collection, data interpretation, editing the manuscript. Eugen Trinka: conceptualization, design, data interpretation, editing the manuscript.

## FUNDING INFORMATION

No funding was received for this study.

## CONFLICT OF INTEREST STATEMENT

Sándor Beniczky received speaker honoraria from Eisai and UCB, not related to this paper. Eugen Trinka has received personal honoraria for lectures and educational activities from EVER Pharma, Marinus, Arvelle, Angelini, Alexion, Argenx, Medtronic, Biocodex, Bial‐Portela & Ca, NewBridge, GL Pharma, GlaxoSmithKline, Boehringer Ingelheim, LivaNova, Eisai, Epilog, UCB, Biogen, Sanofi, STOKE Therapeutics, Jazz Pharmaceuticals, and Rapport; his institution has received research grants from Biogen, UCB Pharma, Eisai, Red Bull, Merck, Bayer, the European Union, FWF Österreichischer Fond zur Wissenschaftsforderung Bundesministerium für Wissenschaft und Forschung, and Jubiläumsfond der Österreichischen Nationalbank. ET is co‐founder and CMO of PrevEp Inc., co‐Director of the European Consortium for Epilepsy Trials (ECET), and Executive Committee member of the Epilepsy Study Consortium, Inc. (ESCI). J. Helen Cross has acted as an investigator for studies with Jazz/GW Pharmaceuticals, Stoke Therapeutics, UCB/Zogenix, Ultragenyx, Encoded, Epigenyx, and Lundbeck; has been a speaker and has served on advisory boards for Biocodex, Jazz Pharmaceuticals, Nutricia, Stoke Therapeutics, Neuraxpharm and UCB (all remuneration has been paid to her department); holds an endowed chair at the University College of London Great Ormond Street Institute of Child Health; has received grants from the National Institute for Health and Care Research (NIHR), the Engineering and Physical Sciences Research Council (EPSRC), the Great Ormond Street Hospital for Children (GOSH) Charity, LifeArc, and Epilepsy Institute UK; and her research is supported by the NIHR Great Ormond Street Hospital Biomedical Research Centre. The remaining authors declare no conflicts of interest.

## CONSENT

The authors have nothing to report.

## Supporting information


Figure S1

## Data Availability

The data that supports the findings of this study are available in the supplementary material of this article.
