# Supplementary material for: Informed acceptance and perceptions of the 2025 ILAE seizure classification following structured teaching
Source: Epileptic Disord. 2026 May 11;28(4):1204–10. doi: 10.1002/epd2.70278 (PMC13499280; doi:10.1002/epd2.70278)

Supplementary material 1

Geographic distribution of participants by country

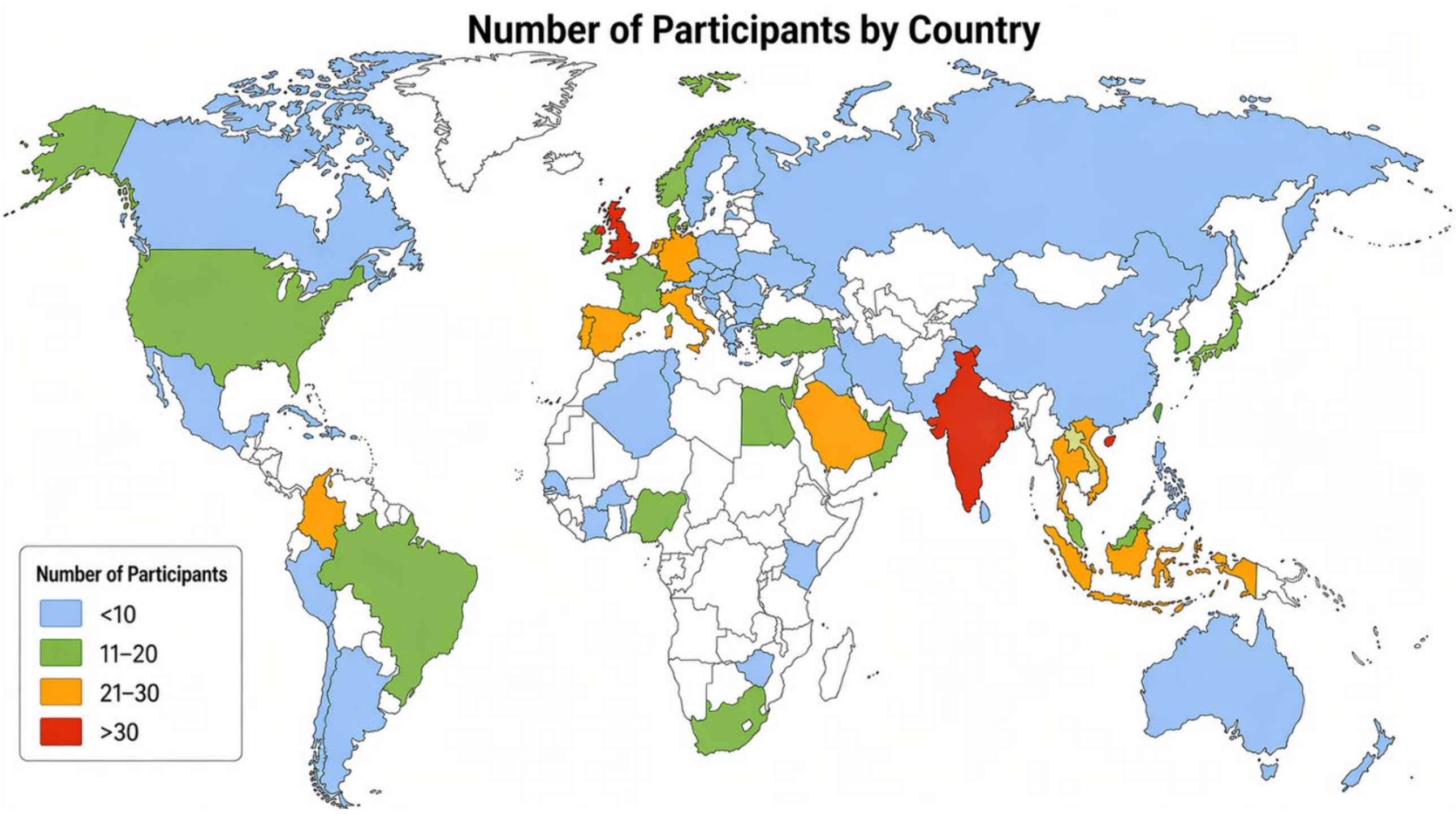

Supplement: Supplementary file 1 [file EPD2-28-1204-s001.pdf]
